# Advantages of Allogeneic Mesenchymal Stem Cells as an Innovative Therapy in Patients with Endometrium Dysfunction

**DOI:** 10.3390/cells15050400

**Published:** 2026-02-26

**Authors:** Tanya N. Timeva, Atanas Shterev, Stanimir Kyurkchiev

**Affiliations:** 1 Head of IVF Department, “D-r Shterev” Ob/Gyn Hospital, 25-31 Hr.Blagoev Str., 1330 Sofia, Bulgaria; ttimeva@yahoo.com (T.N.T.); ashterev@gmail.com (A.S.); 2 Health Department, “A.Kanchev” University of Ruse, 8 Studentska Str., 7017 Ruse, Bulgaria; 3 Tissue Bank “Bulgen”, Hr.Blagoev Str. 25-31, 1330 Sofia, Bulgaria

**Keywords:** thin endometrium, RIF, MSC, scRNA sequencing, angiogenesis, immune regulation

## Abstract

Is it reasonable to use allogeneic mesenchymal stem cells (MSCs) therapy for thin endometrium and recurrent implantation failure? Thin endometrium (TE) and recurrent implantation failure (RIF) are associated with poor reproductive outcomes. Single-cell RNA sequencing (scRNA-seq) shows that such pathologies involve functional impairment of endometrial stromal, vascular, immune cells rather than reductions in cell numbers. MSCs exert regenerative and immunomodulatory effects and are proposed as candidates for endometrial repair. scRNA-seq studies indicate that TE and RIF are characterized by stromal progenitor dysfunction, impaired angiogenesis, immune dysregulation, and cellular senescence, providing a biological rationale for investigating allogeneic MSC-based therapies. scRNA-seq studies of human endometrium in patients with TE and RIF are reviewed alongside experimental and clinical studies evaluating autologous and allogeneic MSCs, with particular emphasis on umbilical cord-derived MSCs. Transcriptomic analyses consistently demonstrate reduced proliferation and decidualization of endometrial stromal cells, suppression of angiogenesis, immune dysregulation, and activation of senescence-associated genes. Preclinical studies show that MSC administration restores endometrial structure, vascularization, and receptivity markers. Early clinical studies suggest potential benefit, although data remain limited and heterogeneous due to non-randomized studies. Allogeneic MSCs are promising as therapy, but further studies on mechanisms and clinical validation are needed.

## 1. Introduction

Infertility remains a major global health concern, affecting approximately 10–15% of couples of reproductive ages. Despite substantial progress in assisted reproductive technologies (ARTs), implantation failure continues to limit overall success rates, underscoring the critical importance of endometrial receptivity in human reproduction. While embryo quality has been extensively studied and refined through advances such as blastocyst culture and preimplantation genetic testing, the endometrium remains a comparatively less tractable determinant of reproductive success. Disorders of endometrial development and function, including thin endometrium (TE), intrauterine adhesions, and recurrent implantation failure (RIF), represent significant unmet clinical needs with limited evidence-based therapeutic options.

The human endometrium is a highly specialized tissue that undergoes cyclical regeneration, differentiation, and shedding under the influence of ovarian steroid hormones. During each menstrual cycle, coordinated interactions among epithelial, stromal, immune, and vascular compartments establish a transient state of receptivity that permits embryo implantation. Disruption of these finely tuned processes may lead to implantation failure even in the presence of morphologically and genetically competent embryos. Increasing evidence indicates that endometrial dysfunction is frequently underdiagnosed and undertreated in clinical practice, contributing to repeated ART failure and substantial emotional and economic burden for affected patients [1,2,3].

Recent advances in transcriptomic technologies, particularly single-cell RNA sequencing (scRNA-seq), have transformed understanding of endometrial biology by enabling high-resolution characterization of individual cell populations and their functional states. These approaches have revealed extensive cellular heterogeneity and dynamic intercellular communication networks that are obscured by bulk analyses. Application of scRNA-seq to pathological conditions such as TE and RIF has uncovered distinct molecular signatures associated with impaired proliferation, angiogenesis, immune regulation, and cellular aging. Parallel developments in regenerative medicine have identified mesenchymal stem cells (MSCs) as potential therapeutic agents capable of modulating these pathological processes through paracrine and immunoregulatory mechanisms.

This review discusses current knowledge on the pathophysiology of thin endometrium and recurrent implantation failure, with a particular focus on insights gained from scRNA-seq studies. We further discuss the biological rationale and emerging clinical evidence supporting MSC-based regenerative therapies, highlighting the potential advantages of umbilical cord-derived MSCs (UC-MSCs) as innovative therapeutic agents in reproductive medicine.

## 2. Endometrium

Until recently, the endometrium was considered to be a passive participant of implantation process while the embryo is the active one which selects the proper site for attachment and invasion in the uterus. On the basis of convincing results from in vitro studies, it is proposed that the endometrium is an active biosensor and determinant of the successful implantation [4].

The endometrium undergoes profound cyclical changes in response to estrogen and progesterone, transitioning from a proliferative to a receptive and ultimately secretory phase. Adequate endometrial development during the peri-implantation period is considered essential for embryo attachment, invasion, and placentation. Among various parameters used to assess endometrial preparedness, endometrial thickness measured by transvaginal ultrasound has been widely adopted in clinical practice for both natural and ART cycles due to its non-invasiveness [5].

### 2.1. Definition and Clinical Significance of Thin Endometrium

Thin endometrium is generally defined as failure of the endometrial lining to achieve sufficient thickness during the late proliferative or early secretory phase, when implantation is expected to occur. Although no universally accepted diagnostic threshold exists, most studies define TE as an endometrial thickness <7 mm, particularly in the context of embryo transfer cycles. Alternative cut-off values ranging from 6 to 8 mm have been reported, reflecting heterogeneity in study designs, stimulation protocols, and patient populations [6]. Despite this variability, an endometrial thickness below these thresholds is consistently associated with impaired reproductive outcomes.

Multiple cohort studies and meta-analyses have demonstrated significantly reduced implantation, clinical pregnancy, and live birth rates in IVF cycles when endometrial thickness is ≤7 mm at the time of ovulation trigger or embryo transfer [7]. Nevertheless, sporadic successful pregnancies have been reported in women with markedly thin endometrium, indicating that thickness alone does not fully capture the biological complexity of endometrial receptivity. In patients with thin endometrium who have conceived, a number of various obstetrical complications have been observed and their list includes early pregnancy loss, miscarriage and ectopic pregnancy, two-fold increase in preterm delivery, low birth weight, and intrauterine growth restriction [8]. These observations highlight the need for more detailed studies on the mechanisms and understanding of TE beyond a simple morphometric assessment.

### 2.2. Etiology and Pathophysiology

The pathophysiology of thin endometrium is multifactorial. Inadequate estrogenic stimulation or impaired estrogen receptor signaling may limit stromal and epithelial proliferation during the follicular phase. Vascular insufficiency, characterized by reduced uterine perfusion and impaired angiogenesis, further compromises endometrial growth. Structural damage to the basal layer of the endometrium, commonly resulting from intrauterine infection, repeated curettage, or adhesions can permanently impair regenerative capacity. Chronic inflammatory states, immune dysregulation, and age-related cellular changes have also been implicated [9]. Thin endometrium can be caused by long-term use of IUD (intrauterine device) containing progesterone as it was established that after 3 months the endometrial thickness and menstrual volume scores were significantly lower than those parameters before the introduction of the IUD [10].

Importantly, these mechanisms do not operate in isolation but interact dynamically within the endometrial microenvironment. Advances in scRNA-seq have provided an unprecedented opportunity to dissect these interactions at cellular resolution.

## 3. scRNA-seq Insights into Thin Endometrium Cells

Collectively, these scRNA-seq findings demonstrate that TE is characterized by intrinsic defects in stromal stem/progenitor cell function rather than simple hormonal insufficiency. An excellent recent review very thoroughly outlines the major alterations in basic functional processes detected by scRNA sequencing in thin endometrium and they include abnormal cellular senescence, altered immune microenvironment, impaired proliferation capability, endometrial dysangiogenesis, endometrial fibrosis, dysfunctional metabolism and receptivity [11].

### 3.1. Impaired Proliferative Capacity of Endometrial Stromal Cells

Endometrial stromal cells constitute approximately 70% of the endometrial mass and are central to cyclic regeneration and decidualization [12]. It is therefore reasonable to assume that the proliferative capacity of stromal stem/progenitor cells largely determines endometrial thickness.

scRNA-seq studies have consistently demonstrated impaired proliferation of stromal cells in TE. Significant downregulation was reported for genes associated with cell cycle progression and proliferation, including RNA polymerase III subunit K, nucleoside diphosphate kinase D, and MKI67 [13]. Further, a marked reduction was detected in proliferating stromal cell populations characterized by high expression of PCNA and cyclin B1 [14]. These findings indicate that insufficient stromal cell expansion is a fundamental feature of TE.

At the signaling level, pathways critical for stromal self-renewal and proliferation are disrupted. NOTCH1–WNT4 signaling, which promotes stromal stem cell maintenance and decidual competence, is significantly suppressed in TE [15]. NOTCH1 expression enhances clonogenicity and modulates stromal differentiation, and its downregulation may critically limit regenerative potential. Similarly, reduced PDGFB–PDGFRB signaling impairs stromal–perivascular interactions essential for tissue growth.

Ligand–receptor interaction analyses reveal additional defects in intercellular communication. Pro-proliferative PTN and TWEAK signaling pathways, normally active in healthy endometrium, are absent in TE, further suppressing stromal expansion [16]. Conversely, upregulation of the inhibitory SEMA3B–NRP1/NRP2 pathway suppresses epithelial–stromal crosstalk and stromal proliferation. Altered dynamics of estrogen and progesterone receptor expression during the menstrual cycle have also been reported, suggesting impaired hormonal responsiveness in TE [15].

### 3.2. Angiogenesis Disorders in Thin Endometrium

Reduced endometrial vascular density is a defining pathological feature of TE. Clinically, TE is associated with decreased VEGF expression and increased uterine artery resistance throughout the menstrual cycle, with endometrial thickness inversely correlated with resistance indices [10].

In the endometrium, mesenchymal stem cells located in perivascular niches play a critical role in angiogenesis by supporting endothelial cell proliferation, vessel stabilization, and tissue remodeling. scRNA-seq studies have revealed profound dysfunction of these perivascular MSCs in TE. Increased synthesis and deposition of type IV collagen around blood vessels, a hallmark of cellular aging, have been detected, resulting in thickened basement membranes and reduced vascular plasticity [14,17].

Ligand–receptor analyses further demonstrate a significant decrease in VEGFA–FLT1 signaling, the principal driver of angiogenesis, alongside increased activity of the angiogenesis-inhibitory SEMA3A–NRP1 pathway [14]. These molecular alterations provide a mechanistic explanation for reduced vessel density, impaired oxygen and nutrient delivery, and limited endometrial growth.

### 3.3. Endometrial Immune Dysregulation

In-depth studies of TE samples by scRNA sequencing have demonstrated significant disturbances in immune cells, with a significant decrease in the concentration of macrophages, natural killer (NK) cells and T cells. Regarding macrophages, a significant overall decrease in their ratio was found, but more importantly, with the increasing use of scRNA sequencing, many new markers of subsets associated with macrophages (other than M1 and M2) have emerged. Some of these macrophage subtypes are associated with the stimulation of fibrosis in various organs such as liver (CD9+), lung (CX3-C-1+ Siglec-F+), and muscle (galectin+). Facts have been published about the presence of a particular subtype of CD301+ macrophages in the stromal cells of the endometrium in samples from patients with TE. It has been shown that CD301+ macrophages are significantly increased and interact with profibrotic cells in the endometrium of patients with TE more actively compared to normal endometrium. The increase in CD301+ macrophages enhances the differentiation of endometrial stromal cells into myofibroblasts and leads to the accumulation of extracellular matrix, which disrupts the physiological architecture of the endometrial tissue, leads to endometrial fibrosis and ultimately leads to female infertility or adverse pregnancy outcomes [18]. These results outline endometrial CD301+ macrophages as a possible target for anti-fibrotic therapy.

scRNA-seq and bulk RNA sequencing studies consistently demonstrate immune dysregulation in TE. Quantitative reductions in macrophages, uterine natural killer (uNK) cells, and T lymphocytes have been reported, alongside qualitative changes in immune activation pathways [19].

Importantly, scRNA-seq has identified expansion of a profibrotic CD301^+^ macrophage subset in TE. These macrophages interact extensively with stromal cells and promote differentiation into myofibroblasts, leading to excessive extracellular matrix deposition and fibrosis [18]. Fibrotic remodeling disrupts normal tissue architecture and compromises receptivity.

NK cells, which represent the dominant immune population during the window of implantation, exhibit altered transcriptional profiles in TE. Bulk transcriptomic analyses reveal increased expression of cytotoxic markers such as GNLY, GZMA, and CORO1A, alongside enrichment of Th1 pro-inflammatory pathways [20,21,22]. These findings suggest impaired immune tolerance and excessive inflammatory signaling, which may hinder implantation.

Although scRNA-seq studies specifically focused on NK cells in TE remain limited, insights from RIF and related implantation disorders provide indirect evidence of disrupted uNK subpopulations and cytokine networks [14,23,24]. Dysregulation of microRNA-mediated control of NK cell function has also been implicated [25].

### 3.4. Cellular Senescence in Thin Endometrium

One of the most distinctive findings from scRNA-seq studies of TE is widespread activation of cellular senescence programs. Cellular senescence is a state of irreversible cell cycle arrest induced by stress inducing factors such as oxidative damage, telomere shortening, and chronic inflammation [26,27,28]. Senescent cells express and secrete growth factors, metalloproteinases and pro-inflammatory cytokines, forming a specific secretory phenotype, defined as senescence-associated secretory phenotype (SASP) or senescence messaging secretome (SMS). The composition of SASP has been shown to contain pro-inflammatory and immunomodulatory cytokines IL6 and IL8, growth factors such as IGFBP, and secreted cell receptors such as TNF receptors. The broad profile of SASP is not affected by the type of inducing agent [26].

While controlled senescence is required for normal decidualization and implantation [27,28], excessive senescence is detrimental. In TE, stromal and perivascular cells exhibit high expression of CDKN2A (p16) and CDKN1A (p21), leading to suppressed proliferation and differentiation [14]. Reduced PTN and EGF signaling further impairs regenerative capacity. These findings implicate premature aging of endometrial progenitor cells as a central mechanism underlying TE.

## 4. Repeated Implantation Failure (RIF)

Repeated implantation failure is commonly defined as failure to achieve a clinical pregnancy following transfer of two or more high-quality embryos across at least two IVF cycles. RIF represents a complex and multifactorial condition involving maternal, embryonic, and uterine factors [29,30]. Recently, it was proposed that repeated implantation failure is a result from the breach of the dynamic interactions between the embryo and the endometrium which otherwise would have initiated a successful implantation and possibly pregnancy [4].

Maternal factors include advanced age, hormonal imbalance, thyroid dysfunction, hyperprolactinemia, and immune dysregulation [31,32,33]. Uterine abnormalities such as fibroids, polyps, congenital anomalies, and adhesions may further impair implantation, and hysteroscopic evaluation is recommended for affected patients [34]. While embryo aneuploidy plays a role, many RIF cases persist despite transfer of euploid embryos, implicating endometrial dysfunction as a major contributor.

### scRNA-seq Insights into RIF

scRNA-seq studies demonstrate that RIF is characterized primarily by qualitative defects in endometrial cell function rather than loss of specific cell populations. Stromal fibroblasts from RIF endometrium exhibit impaired decidualization, with reduced expression of IGFBP1, PRL, FOXO1, and HAND2, indicating defective progesterone responsiveness [14].

Epithelial cells display dysregulation of genes involved in embryo adhesion and signaling, including LIF, SPP1, integrins, and mucins, suggesting impaired epithelial–embryo crosstalk [23,34]. Immune profiling reveals aberrant uNK cell activation and pro-inflammatory macrophage polarization, disrupting trophoblast invasion and vascular remodeling. Endothelial and perivascular cells exhibit altered angiogenic and hypoxia-response signaling [24,35]. These findings reinforce the concept that RIF reflects impaired cellular plasticity and intercellular communication rather than reduced endometrial cellularity.

Refractory thin endometrium is a high challenge for reproductive medicine because it is the cause of poor results of IVF for treatment of infertility. A number of therapies are currently tested as one of them is PRP (platelet rich plasma) injected in the endometrium or just instilled into the uterine cavity. Similarly, injections of G-CSF (granulocyte colony stimulating factor) or growth hormone (GH) got the attention of the gynecologists. Stem cell-based therapies should be included in that list.

## 5. Allogeneic Mesenchymal Stem Cells as an Innovation Therapy

It should be stressed out that molecular and pathway analyses are intended to establish mechanistic plausibility for how MSC therapy could modulate the identified dysregulated pathways, rather than to imply direct causal links to clinical benefit. These findings support a mechanistic rationale for MSC therapy but do not, on their own, establish a causal relationship with clinical efficacy. Next stages for research should be to prove the effects of stem cell therapy for correction of these molecular and cellular alterations in pre-clinical animal studies and ex vivo experiments.

Mesenchymal stem cells (MSCs) have been proposed as an investigational therapy aimed at restoring endometrial function through regenerative and immunomodulatory mechanisms.

MSCs are defined by their adherence to plastic, expression of specific surface markers, and capacity for multilineage differentiation, in accordance with the International Society for Cell & Gene Therapy criteria [36]. Bone marrow-derived MSCs (BM-MSCs), and adipose-derived MSCs (AD-MSCs) represent major sources of MSCs investigated for therapeutic applications, yet they exhibit distinct biological and translational characteristics that inform their clinical utility. BM-MSCs have historically served as a gold standard, particularly for skeletal tissue repair due to their robust osteogenic and chondrogenic differentiation potential, but their clinical translation is constrained by invasive harvesting procedures, limited proliferative capacity, and age-related functional decline [37]. In contrast, AD-MSCs offer higher initial yields and less invasive procurement than BM-MSCs, with useful multipotent differentiation and immunomodulatory capabilities suitable for soft-tissue regeneration; however, donor variability can contribute to heterogeneity across batches [38]. UC-MSCs, harvested non-invasively from perinatal tissue that is typically discarded at birth, combine ethical ease with a more primitive phenotype characterized by enhanced proliferative capacity, delayed senescence, and reduced donor heterogeneity relative to adult-tissue MSCs [39].

Mesenchymal stem cells have emerged as promising candidates for regenerative therapy in refractory TE and selected cases of RIF. MSCs exert therapeutic effects primarily through paracrine mechanisms, secreting growth factors and immunomodulatory cytokines that promote angiogenesis, suppress inflammation, reduce fibrosis, and enhance endogenous tissue repair [40,41].

Multiple sources of MSCs have been evaluated in the context of thin endometrium and RIF. Bone marrow-derived MSCs remain the most extensively studied and have been used in patients with severe endometrial damage, including intrauterine adhesions and Asherman syndrome [42,43]. However, given the invasive nature of bone marrow harvesting, alternative sources such as menstrual blood-derived MSCs, umbilical cord-derived MSCs, and adipose-derived MSCs have been investigated. Menstrual blood-derived MSCs are of particular interest due to their non-invasive collection, autologous use, and intrinsic endometrial affinity, while UC-MSCs offer high proliferative capacity and low immunogenicity in an allogeneic setting [44,45].

Clinical evidence supporting MSC-based therapy for refractory thin endometrium is currently limited to small prospective studies, pilot trials, and case series. These studies report increases in endometrial thickness, improvements in endometrial vascularity, restoration of menstrual function, and subsequent embryo implantation following intrauterine infusion or hysteroscopic administration of MSCs [46,47,48].

Similarly, in selected patients with suspected endometrial-related RIF, MSC administration has been associated with improved implantation and clinical pregnancy rates. However, consistent with ESHRE and ASRM positions on ART add-ons, the quality of evidence remains low, with substantial heterogeneity in patient selection, cell source, dosage, timing of administration, and outcome measures.

From a safety perspective, available studies suggest a favorable short-term safety profile, with no serious adverse events, immune reactions, or malignant transformation reported to date [44,45]. Nonetheless, both ESHRE and ASRM emphasize that interventions lacking robust evidence from randomized controlled trials should be considered experimental, and patients should be appropriately counseled regarding the uncertainty of benefits, potential risks, and regulatory considerations.

## 6. Advantages of UC-MSCs as Therapeutic Agents

Allogeneic MSCs offer distinct advantages over autologous cell sources, including immediate availability, consistent quality, high proliferative capacity, and reduced donor variability. UC-MSCs/WJ-MSCs, exhibit low expression of HLA class II molecules and costimulatory factors, conferring low immunogenicity and enabling safe intrauterine application without immunosuppression [47,48]. These properties make allogeneic MSCs particularly suitable for reproductive indications requiring timely intervention (Table 1).

Experimental evidence indicates that the therapeutic effects of MSCs in thin endometrium and RIF are mediated predominantly through paracrine signaling rather than durable engraftment. In vitro studies have demonstrated that WJ-MSCs protect damaged human endometrial stromal cells from apoptosis and enhance cellular proliferation via the secretion of growth factors and anti-inflammatory cytokines [49]. Additional studies have shown that WJ-MSCs can be induced to differentiate into endometrial-like epithelial and stromal cells under defined conditions, supporting their regenerative potential [50].

Animal models further support these findings. In rodent models of thin endometrium, allogeneic UC-MSC administration resulted in increased endometrial thickness, restoration of glandular architecture, enhanced vascularization, and improved implantation rates. MSC-based therapy was also associated with reduced endometrial fibrosis and upregulation of endometrial receptivity markers, including HOXA10 and leukemia inhibitory factor (LIF), which are critical for embryo implantation [51,52]. These mechanisms directly address key pathological features implicated in RIF.

Still, the results from pre-clinical studies are not a direct evidence of clinical efficacy, where applicable, we reference existing preclinical proof-of-concept studies supporting these mechanisms, while clearly stating that direct evidence of clinical efficacy cannot be inferred from the present data.

Clinical translation of allogeneic MSC therapy has focused primarily on women with refractory thin endometrium, frequently in the context of Asherman syndrome. Prospective clinical studies have demonstrated that intrauterine transplantation of allogeneic UC-MSCs delivered via a collagen scaffold significantly increases endometrial thickness and improves receptivity markers in patients unresponsive to standard treatments [49]. Importantly, successful clinical pregnancies and live births have been reported following MSC therapy in this population.

More recently, a randomized controlled clinical trial evaluating collagen scaffold-based delivery of allogeneic UC-MSCs demonstrated a trend toward improved cumulative clinical pregnancy and live birth rates compared with control treatment, further supporting the therapeutic potential of this approach. Scaffold-assisted delivery appears to enhance MSC retention within the uterine cavity and prolong local paracrine effects, addressing a major limitation of direct intrauterine injection [48].

Although few studies have specifically enrolled patients with RIF as a primary diagnosis, the biological effects observed following MSC therapy directly target the molecular and structural abnormalities associated with implantation failure. Improvements in angiogenesis, stromal cell function, and expression of receptivity markers following MSC treatment suggest a strong mechanistic rationale for extending allogeneic MSC therapy to RIF patients, particularly those with persistently thin or poorly responsive endometrium. 

Despite encouraging results, existing clinical data are limited by small sample sizes, heterogeneity in MSC preparation, delivery methods, and outcome definitions. A recent preclinical meta-analysis confirmed consistent benefits of MSC therapy on endometrial thickness, receptivity, and pregnancy outcomes in animal models, supporting biological plausibility but underscoring the need for well-powered human trials [53].

Despite promising preclinical and early clinical findings, MSC-based therapies for thin endometrium and recurrent implantation failure (RIF) still face several important challenges that currently limit their clinical implementation. Major difficulties are the optimal stem cell source, dose, delivery route (intrauterine infusion, subendometrial injection). Timing of application relative to the implantation window remains insufficiently standardized, leading to variability in clinical outcomes. Additionally, the mechanisms of action whether via direct engraftment, paracrine signaling, immunomodulation, or endometrial niche restoration, are not fully elucidated, complicating protocol optimization and regulatory evaluation. Safety concerns, including the potential for aberrant differentiation, ectopic tissue formation, fibrosis, or long-term oncogenic risk, require rigorous long-term follow-up, which is currently lacking in most studies. Finally, regulatory, manufacturing, and cost barriers associated with advanced therapy medicinal products (ATMPs) pose significant translational challenges. Collectively, these factors highlight the need for well-designed randomized controlled trials, standardized manufacturing and delivery protocols, and mechanistic studies to establish stem cell therapy as a reliable clinical option for thin endometrium and RIF. Future studies should focus on standardized MSC characterization, optimized dosing strategies, and randomized trials with implantation and live birth as primary endpoints, particularly in RIF populations.

Stem cell therapy for thin endometrium or RIF should be considered as Advanced Therapy Medicinal Products (ATMPs) which are personalized and based on “living” cells. Any trial with patients with thin endometrium or RIF should follow strictly the regulations of the European Medicines Agency (AMA).

### 6.1. MSC Exosomes as Therapy for Thin Endometrium

Exosomes are nano vesicles (30–150 nm) limited by a lipid bilayer and specific contents, which are secreted into extracellular space and serve as mediators of intracellular interactions. These vesicles transport various factors to far distances in the organism and react with the target cells either via internalization or ligand/receptor interactions. Exosomes mediate intercellular communication as cell-derived extracellular signaling organelles that transmit specific information from their cell of origin to their target cells.

Secretion of proteins and nucleic acids through exosomes confers specific features and advantages to this process such as 3D structure and biological role of the cargo molecules in exosomes; direct cell-to-cell contact is not needed for delivery of molecular signals; concentration of specific proteins inside exosomes is maintained at high levels; accurate delivery of biomolecules to the target cell is assured by specific surface receptors and can be achieved at long distance between the cells; no necessary de novo secretion of delivered factors in the target cell.

While mesenchymal stem cell (MSC) therapy has shown encouraging potential for the treatment of thin endometrium (TE) and recurrent implantation failure (RIF), growing evidence suggests that many of its therapeutic benefits are mediated predominantly through paracrine mechanisms rather than long-term cell engraftment. In this context, MSC-derived exosomes have emerged as an attractive cell-free alternative that may overcome several limitations associated with live cell therapies.

Exosomes contain bioactive proteins, lipids, and regulatory RNAs that mediate the pro-angiogenic, anti-fibrotic, anti-apoptotic, and immunomodulatory effects of parent MSCs—key processes implicated in endometrial regeneration and receptivity.

Compared with whole MSCs, exosome-based therapies offer a superior safety profile by eliminating risks related to uncontrolled cell proliferation, ectopic differentiation, and potential long-term oncogenicity, which are particularly relevant concerns in reproductive tissues requiring repeated intrauterine interventions (Table 1). Moreover, the nanoscale size of exosomes facilitates efficient penetration into poorly vascularized or fibrotic endometrial tissue, a hallmark of severe TE, where cell engraftment may be limited. Their low immunogenicity and absence of major histocompatibility complex molecules further support their suitability for allogeneic, off-the-shelf use in immunologically sensitive implantation settings.

From a translational perspective, MSC exosomes also present notable advantages in terms of manufacturing consistency, storage stability, and dosing standardization, addressing key regulatory and logistical challenges associated with advanced therapy medicinal products (ATMPs). Importantly, exosomes can be engineered or preconditioned to enhance cargo relevant to endometrial repair and implantation, offering opportunities for precision medicine approaches tailored to distinct TE and RIF phenotypes. Collectively, these attributes position MSC-derived exosomes as a promising next-generation therapeutic modality that may complement or, in some contexts, supersede cell-based MSC therapy for improving endometrial receptivity and reproductive outcomes (Table 2).

MSC-derived exosomes retain the regenerative and immunomodulatory efficacy of MSCs while offering superior safety, scalability, and translational feasibility, making them particularly well suited for the treatment of thin endometrium and recurrent implantation failure.

#### 6.1.1. Preclinical Evidence in Endometrial Repair

**Figure 1:**
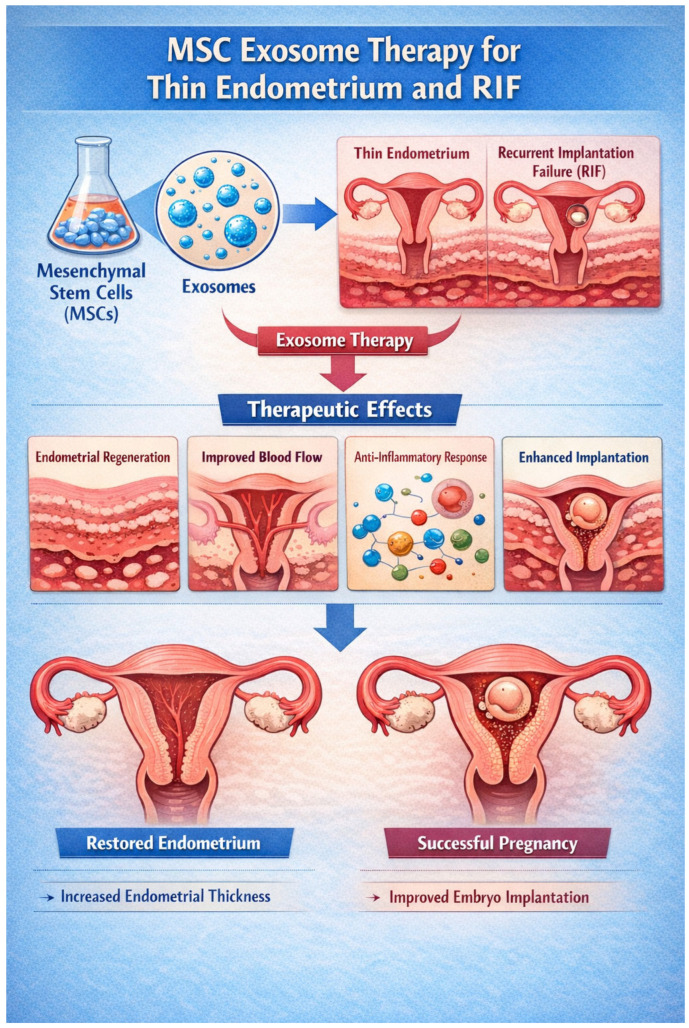
MSC-derived exosome therapy in thin endometrium and recurrent implantation failure (RIF). MSC-derived exosomes deliver regulatory RNAs, proteins, and growth factors that promote endometrial regeneration, angiogenesis, and immunomodulation. These effects increase endometrial thickness, improve uterine receptivity, and enhance embryo implantation, supporting successful pregnancy outcomes.

Animal models of thin endometrium confirm the regenerative potential of MSC-Exos. In a rat model of thin endometrium, intrauterine application of human umbilical cord MSC-exosome gel significantly improved endometrial thickness, increased gland number, enhanced subendometrial microangiogenesis, reduced fibrosis, and increased pregnancy rates compared with controls. Importantly, exosome treatment outperformed MSCs alone in inhibiting fibrosis through modulation of TGF-β1/Smad2/3 signaling (Figure 1). In addition, exosomes accelerated early endometrial repair by regulating ECM remodeling via miR-202-3p, which suppressed MMP11 and increased collagen and fibronectin deposition, contributing to early structural restoration in injured tissue. This body of preclinical evidence supports the mechanistic basis for clinical translation of MSC-Exos in enhancing structural and functional aspects of endometrial regeneration [54].

Preclinical studies reveal that MSC-Exos enhance endometrial cell proliferation and viability, suppress apoptosis, and modulate key signaling pathways involved in tissue repair. In vitro, exosomes derived from human umbilical cord MSCs (hUCMSC-Exos) promoted endometrial stromal cell proliferation and protected against induced injury via activation of autophagy and anti-apoptotic pathways such as PTEN/AKT [55]. Similarly, exosomes loaded with specific miRNAs (e.g., miR-99b-5p) enhanced proliferation and reduced apoptosis in damaged endometrial stromal cells by targeting PCSK9, indicating molecular specificity in their reparative action [56].

Beyond structural improvement, MSC-Exos mitigate pathological fibrosis that is a key barrier to functional endometrial recovery. In vitro studies show that MSC-Exos reverse TGF-β1-induced fibrosis in endometrial epithelial cells by altering their miRNA profile, activating P62-dependent autophagy, and improving cell proliferation and survival, all necessary for a receptive endometrial environment [57].

#### 6.1.2. Clinical Perspectives and Current Limitations

Data from preclinical and ex vivo studies show that MSC exosomes can be considered as biological carriers of active substances that improve the functional potential of damaged endometrium and can be an alternative of MSC-based therapies. Further investigations on the possibility to use MSC-exosomes as cell-free therapies for correction of endometrium dysfunction could be a very promising direction of clinical research and trials [58]. However, clinical evidence specifically for MSC-exosome therapy in human thin endometrium or RIF remains limited, with most clinical studies yet to be conducted. Small early phase or pilot clinical explorations and registered trials may incorporate exosome-based approaches in the near future, but robust randomized controlled trials are not yet widely reported.

A major barrier to clinical adoption is the variability in exosome isolation, characterization, and dosage standardization. Techniques such as ultrafiltration combined with size exclusion chromatography are proposed to yield high-purity exosomes with retained biological activity, but further optimization and regulatory standardization remain necessary before broad clinical implementation.

Optimal dosing regimens (concentration, frequency), route of administration (intrauterine vs. systemic), and application timing relative to ART cycles are not yet established. Consistent protocols could improve comparability between studies and accelerate clinical translation.

Well-designed, adequately powered randomized controlled trials are needed to validate safety and efficacy in infertile patients with thin endometrium and RIF. Biomarkers of receptivity and implantation outcomes should be incorporated to clarify mechanisms of benefit in humans.

MSC-derived exosomes represent a promising cell-free regenerative therapy for addressing thin endometrium and possibly improving implantation outcomes in RIF. Preclinical studies demonstrate that exosomes enhance endometrial proliferation, reduce fibrosis, and improve functional markers of receptivity via molecular cargo delivery (e.g., miRNAs) that modulate apoptosis, autophagy, and ECM remodeling. Despite encouraging mechanistic and animal data, clinical evidence is currently limited, warranting larger controlled human trials and standardized therapeutic protocols. If validated, MSC-exosomes could become an innovative adjunct in ART, offering targeted endometrial repair with favorable safety profiles.

## 7. Conclusions

Thin endometrium and recurrent implantation failure represent major, unresolved barriers to successful assisted reproduction, increasingly recognized as disorders of endometrial regeneration, cellular plasticity, and intercellular communication rather than simple hormonal insufficiency. Advances in single-cell transcriptomics have fundamentally reshaped our understanding of these conditions, revealing coordinated defects in stromal progenitor proliferation, angiogenesis, immune regulation, and cellular senescence that together compromise endometrial receptivity and embryo–endometrium crosstalk.

Within this mechanistic framework, mesenchymal stem cell-based therapies have emerged as a biologically rational regenerative approach. Preclinical and early clinical studies indicate that MSCs and particularly umbilical cord-derived MSCs—can partially restore endometrial structure and function through paracrine, anti-fibrotic, pro-angiogenic, and immunomodulatory effects. However, variability in cell sources, delivery strategies, and clinical endpoints currently limits widespread clinical adoption.

MSC-derived exosomes represent a promising next-generation, cell-free alternative that may overcome key limitations of live cell therapy. By delivering defined molecular cargoes that directly target angiogenesis, fibrosis, inflammation, and cellular aging, exosomes retain the therapeutic benefits of MSCs while offering improved safety, scalability, and regulatory feasibility. Robust preclinical evidence supports their capacity to enhance endometrial repair and receptivity, yet high-quality clinical data in thin endometrium and RIF remain limited.

Future progress will depend on standardized exosome manufacturing and characterization, optimization of dosing and delivery strategies, and well-designed randomized controlled trials with implantation and live birth as primary outcomes. If these challenges are addressed, MSC-derived exosomes may become a transformative adjunct in assisted reproductive technologies, enabling targeted restoration of endometrial function and improved reproductive outcomes in patients with otherwise refractory implantation failure.

## Figures and Tables

**Table 1:** Comparative characteristics of mesenchymal stem cells derived from umbilical cord, bone marrow, and adipose tissue.

Feature	UC-MSCs	BM-MSCs	AD-MSCs
Tissue source	Umbilical cord (Wharton’s jelly)	Bone marrow aspirate	Adipose tissue
Donor age	Neonatal	Adult	Adult
Harvesting procedure	Non-invasive	Invasive	Minimally invasive
Ethical concerns	Minimal	Minimal	Minimal
Initial cell yield	Moderate-high	Low	High
Proliferative capacity	High	Low-moderate	Moderate
Senescence onset	Delayed	Early	Intermediate
Telomere length	Long	Short	Intermediate
Donor variability	Low	High	High
Immunogenicity (HLA-II)	Very low	Inducible	Inducible
Allogeneic suitability	Excellent	Moderate	Moderate
Immunomodulatory potency	Strong	Strong	Moderate
Paracrine/secretory activity	High	Moderate	High
Angiogenic potential	High	Moderate	High
Osteogenic differentiation	Moderate	High	Moderate
Chondrogenic differentiation	Moderate-high	High	Moderate
Adipogenic differentiation	Low	Moderate	High
Manufacturing scalability	High	Limited	Moderate
Off-the-shelf potential	High	Moderate	Moderate
Primary clinical strengths	Immunomodulation, allogeneic therapy	Bone/cartilage repair	Soft tissue repair

**Table 2:** Comparison of therapies with MSCs or MSC-derived exosomes in thin endometrium and RIF.

Feature	MSC Therapy	MSC-Derived Exosomes
Therapeutic nature	Live cell-based therapy	Cell-free biological therapy
Primary mechanism	Paracrine + limited engraftment	Paracrine signaling only
Safety concerns	Risk of ectopic engraftment, aberrant differentiation, long-term persistence	Minimal tumorigenicity; no engraftment risk
Immunogenicity	Low but inducible under inflammation	Very low; MHC-free
Tissue penetration	Limited in fibrotic/thin endometrium	High (nano-sized vesicles)
Suitability for repeated dosing	Limited by cell viability and safety	Highly suitable
Manufacturing consistency	Donor- and batch-dependent	More standardized and scalable
Storage and stability	Cryopreservation affects viability	Stable; amenable to long-term storage
Regulatory complexity	ATMP (cell therapy)	Lower regulatory burden
Engineering potential	Limited	High (cargo enrichment, preconditioning)
Clinical relevance to TE/RIF	Improves thickness and receptivity in early trials	Targets angiogenesis, fibrosis, inflammation, receptivity

## Data Availability

No new data were created or analyzed in this study.

## References

[1] Ticconi C., Di Simone N., Campagnolo L., Fazleabas A. (2021). Clinical consequences of defective decidualization. Tissue Cell.

[2] Lucan M., Sandor M., Bodog A., Mocuta D., Aur C.D., Sachelarie L., Huniadi A. (2025). Chronic Endometritis: A Silent Contributor to Infertility and Reproductive Failure—A Comprehensive Review. Reprod. Med..

[3] Bastías C.S., Dean M., Luetkemeyer C.M. (2026). Unexplained infertility and impaired decidualization: A case for studying endometrial mechanics and microstructure. Acta Biomater..

[4] Macklon N. (2025). Resolving recurrent implantation failure. Reprod. BioMed. Online.

[5] Timeva T. (2025). Endometrial assessment to ensure successful implantation. Fertility Preservation—Theory and Practice Experience.

[6] Kasius A., Smit J.G., Torrance H.L., Eijkemans M.J.C., Mol B.W., Opmeer B.C., Broekmans F.J. (2014). Endometrial thickness and pregnancy rates after IVF: A systematic review and meta-analysis. Hum. Reprod. Update.

[7] Liu K.E., Hartman M., Hartman A., Luo Z.-C., Mahutte N. (2018). The impact of a thin endometrial lining on fresh and frozen-thaw IVF outcomes. Hum. Reprod..

[8] Mouhayar Y., Franasiak J.M., Sharara F.I. (2019). Obstetrical complications of thin endometrium in assisted reproductive technologies: A systematic review. J. Assist. Reprod. Genet..

[9] Miwa I., Tamura H., Takasaki A., Yamagata Y., Shimamura K., Sugino N. (2009). Pathophysiologic features of thin endometrium. Fertil. Steril..

[10] Yu Y., Zhou Z., Wang L., Liu J. (2022). Effect of Mirena Intrauterine Device on Endometrial Thickness, Quality of Life Score, and Curative Effect in Patients with Perimenopausal Abnormal Uterine Bleeding. Comput. Math. Methods Med..

[11] Zhang X., Lv H., Weng Q., Jiang P., Dai C., Zhao G., Hu Y. (2025). Thin endometrium at single-cell resolution. Am. J. Obstet. Gynecol..

[12] Queckbörner S., von Grothusen C., Boggavarapu N.R., Francis R.M., Davies L.C., Gemzell-Danielsson K. (2021). Stromal heterogeneity in the human proliferative endometrium—A single-cell RNA sequencing study. J. Pers. Med..

[13] Zong L., Zheng S., Meng Y., Tang W., Li D., Wang Z., Tong X., Xu B. (2021). Integrated Transcriptomic Analysis of the miRNA–mRNA Interaction Network in Thin Endometrium. Front. Genet..

[14] Lv H., Zhao G., Jiang P., Wang H., Wang Z., Yao S., Zhou Z., Wang L., Liu D., Deng W. (2022). Deciphering the endometrial niche of human thin endometrium at single-cell resolution. Proc. Natl. Acad. Sci. USA.

[15] Zhang X., Li Y., Chen X., Jin B., Shu C., Ni W., Jiang Y., Zhang J., Ma L., Shu J. (2022). Single-cell transcriptome analysis of thin endometrium. FASEB J..

[16] Xu L., Fan Y., Wang J., Shi R. (2022). Dysfunctional intercellular communication and metabolic signaling pathways in thin endometrium. Front. Physiol..

[17] Masuda H., Anwar S.S., Bühring H.-J., Rao J.R., Gargett C.E. (2012). A novel marker of human endometrial mesenchymal stem-like cells. Cell Transplant..

[18] Lv H., Sun H., Wang L., Yao S., Liu D., Zhang X., Pei Z., Zhou J., Wang H., Dai J. (2023). Targeting CD301^+^ macrophages inhibits endometrial fibrosis and improves pregnancy outcome. EMBO Mol. Med..

[19] Maekawa R., Taketani T., Mihara Y., Sato S., Okada M., Tamura I., Jozaki K., Kajimura T., Asada H., Tamura H. (2017). Thin endometrium transcriptome analysis reveals a potential mechanism of implantation failure. Reprod. Med. Biol..

[20] Lai Z.-Z., Wang Y., Zhou W.-J., Liang Z., Shi J.-W., Yang H.-L., Xie F., Chen W.-D., Zhu R., Zhang C. (2022). Single-cell transcriptome profiling of the human endometrium of patients with recurrent implantation failure. Theranostics.

[21] Niu Y., Le A. (2025). Immune-related gene signatures of thin endometrium: A transcriptomic and single-cell study. Front. Endocrinol..

[22] Vento-Tormo R., Efremova M., Botting R.A., Turco M.Y., Vento-Tormo M., Meyer K.B., Park J.-E., Stephenson E., Polański K., Goncalves A. (2018). Single-cell reconstruction of the early maternal–fetal interface in humans. Nature.

[23] Wang W., Vilella F., Alama P., Moreno I., Mignardi M., Isakova A., Pan W., Simon C., Quake S.R. (2020). Single-cell transcriptomic atlas of the human endometrium during the menstrual cycle. Nat. Med..

[24] Zhang H., Zhang C., Zhang S. (2024). Single-cell RNA transcriptome of the human endometrium reveals epithelial characterizations associated with recurrent implantation failure. Adv. Biol..

[25] Li L., Feng T., Zhou W., Liu Y., Li H. (2021). miRNAs in decidual NK cells: Regulators worthy of attention during pregnancy. Reprod. Biol. Endocrinol..

[26] Chaib S., Tchkonia T., Kirkland J.L. (2022). Cellular senescence and senolytics: The path to the clinic. Nat. Med..

[27] Brighton P.J., Maruyama Y., Fishwick K., Vrljicak P., Tewary S., Fujihara R., Muter J., Lucas E.S., Yamada T., Woods L. (2017). Clearance of senescent decidual cells by uterine natural killer cells in cycling human endometrium. eLife.

[28] Deryabin P., Griukova A., Nikolsky N., Borodkina A.V. (2020). The link between endometrial stromal cell senescence and decidualization in female fertility: The art of balance. Cell. Mol. Life Sci..

[29] Coughlan C., Ledger W., Wang Q., Liu F., Demirol A., Gurgan T., Cutting R., Ong K., Sallam H., Li T.C. (2014). Recurrent implantation failure: Definition and management. Reprod. BioMed. Online.

[30] Timeva T., Shterev A., Kyurkchiev S. (2014). Recurrent implantation failure: The role of the endometrium. J. Reprod. Infertil..

[31] Opuchlik K., Pankiewicz K., Pierzyński P., Sierdziński J., Aleksejeva E., Salumets A., Issat T., Laudański P. (2025). Factors influencing endometrial receptivity in women with RIF. BMC Womens Health.

[32] Herman T., Csehely S., Orosz M., Bhattoa H.P., Deli T., Török P., Lagana A.S., Chiantera V., Jakab A. (2022). Impact of endocrine disorders on IVF outcomes: Results from a large, single-centre, prospective study. Reprod. Sci..

[33] El-Toukhy T., Campo R., Khalaf Y., Tabanelli C., Gianaroli L., Gordts S.S., Mestdagh G., Mardesic T., Voboril J., Marchino G.L. (2016). Hysteroscopy in recurrent in-vitro fertilisation failure (TROPHY): A multicentre, randomised controlled trial. Lancet.

[34] Garcia-Alonso L., Handfield L.-F., Roberts K., Nikolakopoulou K., Fernando R.C., Gardner L., Woodhams B., Arutyunyan A., Polanski K., Hoo R. (2021). Mapping the temporal and spatial dynamics of the human endometrium in vivo and in vitro. Nat. Genet..

[35] Tempest N., Soul J., Hill C.J., Hapangama D.K. (2025). Cell type and region-specific transcriptional changes in the endometrium of women with RIF identify potential treatment targets. Proc. Natl. Acad. Sci. USA.

[36] Dominici M., Le Blanc K., Mueller I., Slaper-Cortenbach I., Marini F.C., Krause D.S., Deans R.J., Keating A., Prockop D.J., Horwitz E.M. (2006). Minimal criteria for defining multipotent mesenchymal stromal cells. The International Society for Cellular Therapy position statement. Cytotherapy.

[37] Song Y., Lim J.Y., Lim T., Im K.I., Kim N., Nam Y.S., Jeon Y.W., Shin J.C., Ko H.S., Park I.Y. (2020). Human mesenchymal stem cells derived from umbilical cord and bone marrow exert immunomodulatory effects in different mechanisms. World J. Stem Cells.

[38] Maldonado V.V., Patel N.H., Smith E.E., Barnes C.L., Gustafson M.P., Rao R.R., Samsonraj R.M. (2023). Clinical utility of mesenchymal stem/stromal cells in regenerative medicine and cellular therapy. J. Biol. Eng..

[39] Galipeau J., Sensébé L. (2018). Mesenchymal stromal cells: Clinical challenges and therapeutic opportunities. Cell Stem Cell.

[40] Caplan A.I., Dennis J.E. (2006). Mesenchymal stem cells as trophic mediators. J. Cell. Biochem..

[41] Gnecchi M., Zhang Z., Ni A., Dzau V.J. (2008). Paracrine mechanisms in adult stem cell signaling and therapy. Circ. Res..

[42] Nagori C.B., Panchal S.Y., Patel H. (2011). Endometrial regeneration using autologous adult stem cells followed by conception by in vitro fertilization in a patient of severe Asherman’s syndrome. J. Hum. Reprod. Sci..

[43] Singh N., Shekhar B., Mohanty S., Kumar S., Seth T., Girish B. (2020). Autologous bone marrow-derived stem cell therapy for Asherman’s syndrome and endometrial atrophy: A 5-year follow-up study. J. Hum. Reprod. Sci..

[44] Nagamura-Inoue T., He H. (2014). Umbilical cord-derived mesenchymal stem cells: Their advantages and potential clinical utility. World J. Stem Cells.

[45] Tan J., Li P., Wang Q., Li Y., Li X., Zhao D., Xu X., Kong L. (2016). Autologous menstrual blood–derived stromal cells transplantation for severe Asherman’s syndrome. Hum. Reprod..

[46] Santamaria X., Cabanillas S., Cervelló I., Arbona C., Raga F., Ferro J., Palmero J., Remohí J., Pellicer A., Simón C. (2016). Autologous cell therapy with CD133^+^ bone marrow-derived stem cells for refractory Asherman’s syndrome and endometrial atrophy: A pilot cohort study. Hum. Reprod..

[47] Zhang Y., Shi L., Lin X., Zhou F., Xin L., Xu W., Yu H., Li J., Pan M., Pan Y. (2021). Unresponsive thin endometrium caused by Asherman syndrome treated with umbilical cord mesenchymal stem cells on collagen scaffolds: A pilot study. Stem Cell Res. Ther..

[48] Cao Y., Sun H., Zhu H., Zhu X., Tang X., Yan G., Wang J., Bai D., Wang J., Wang L. (2018). Allogeneic cell therapy using umbilical cord MSCs on collagen scaffolds for patients with recurrent uterine adhesion: A phase I clinical trial. Stem Cell Res. Ther..

[49] Yang X., Zhang M., Zhang Y., Li W., Yang B. (2011). Mesenchymal stem cells derived from Wharton jelly of the human umbilical cord ameliorate damage to human endometrial stromal cells. Fertil. Steril..

[50] Cao Y., Sun H., Zhu H., Zhu X., Tang X., Yan G., Wang J., Bai D., Wang J., Wang L. (2018). Allogeneic cell therapy using human umbilical cord mesenchymal stem cells for endometrial regeneration: Differentiation into endometrial cells in vitro. Stem Cell Res. Ther..

[51] Rungsiwiwut R., Virutamasen P., Pruksananonda K. (2021). Mesenchymal stem cells for restoring endometrial function: Aninfertility perspective. Reprod. Med. Biol..

[52] Liu Y.Y., Koubanani Z.G., Jia D.Z., Ma M. (2025). Mesenchymal stem cell therapy for endometrial injury: A meta-analysis of preclinical studies. BMC Womens Health.

[53] Zhang S., Wang D., Yang F., Shen Y., Li D., Deng X. (2022). Intrauterine Injection of Umbilical Cord Mesenchymal Stem Cell Exosome Gel Significantly Improves the Pregnancy Rate in Thin Endometrium Rats. Cell Transplant..

[54] Wang J., Hu R., Xing Q., Feng X., Jiang X., Xu Y., Wei Z. (2020). Exosomes Derived from Umbilical Cord Mesenchymal Stem Cells Alleviate Mifepristone-Induced Human Endometrial Stromal Cell Injury. Stem Cells Int..

[55] Li L.F., An J., Wang Y., Liu L., Wang Y., Zhang X.H. (2024). Exosomes Derived from Mesenchymal Stem Cells Increase the Viability of Damaged Endometrial Cells via the miR-99b-5p/PCSK9 Axis. Stem Cells Dev..

[56] Zhou L., Dong L., Li H., Liu H., Yang J., Huang Z. (2023). Mesenchymal stem cell-derived exosomes ameliorate TGF-β1-induced endometrial fibrosis by altering their miRNA profile. Am. J. Transl. Res..

[57] Tabeeva G., Silachevn D., Vishnyakova P., Asaturova A., Fatkhudinov T., Smetnik A., Dumanovskaya M. (2023). The Therapeutic Potential of Multipotent Mesenchymal Stromal Cell—Derived Extracellular Vesicles in Endometrial Regeneration. Int. J. Mol. Sci..

[58] Mariadas H., Chen J.-H., Chen K.-H. (2026). Applications of Exosomes in Female Medicine: A Systematic Review of Molecular Biology, Diagnostic and Therapeutic Perspectives. Int. J. Mol. Sci..

